# Immunohistochemical expression of parathyroid hormone-related protein and ezrin in invasive breast carcinoma of no special type: a retrospective analysis

**DOI:** 10.1186/s13000-025-01598-2

**Published:** 2025-01-18

**Authors:** Menna Allah Gamil Ali Shalaby, Marwa Mohammed Dawoud, Marwa Salah Gadallah, Asmaa Gaber Abdou

**Affiliations:** Department of Pathology, Faculty of Medicine, Menoufia University, Menoufia, Egypt

**Keywords:** Breast carcinoma, Ezrin, Immunohistochemistry, Parathyroid hormone-related protein (PTHrP)

## Abstract

**Background:**

Globally, breast cancer ranks among the most common malignancies and has a high mortality rate. Invasive breast carcinoma of no special type (IBC-NST) presents a heterogeneous group with variable prognosis. Identifying reliable biomarkers is crucial for improving treatment strategies and predicting outcomes. This study investigates the immunohistochemical expression of parathyroid hormone-related protein (PTHrP) and ezrin in IBC-NST and their correlation with clinicopathological features and overall survival.

**Methods:**

This retrospective study analyzed 160 paraffin-embedded tissue samples, including 123 IBC-NST and 37 normal breast tissues, collected from patients treated at Menoufia University Hospital during the period from January 2018 to January 2022. Immunohistochemical staining for PTHrP and ezrin was performed, and expression levels were quantified using the H score.

**Results:**

PTHrP expression was significantly higher in IBC-NST than in adjacent DCIS and normal tissues (*p* < 0.001). High PTHrP percent of expression was associated with metastasis (*p* = 0.009), bone metastasis (*p* = 0.012), and lymphovascular invasion (*p* = 0.037). Ezrin expression was also significantly elevated in IBC-NST, with higher H score values correlating with high tumor grade (*p* = 0.002), high N stage (*p* = 0.045), advanced AJCC stage grouping (*p* = 0.0043) and metastasis (*p* = 0.001). A significant positive correlation was observed between PTHrP and ezrin expression (rs = 0.341, *p* < 0.001). Kaplan-Meier analysis showed that high ezrin expression, in terms of intensity (*p* = 0.007) and H score (*p* = 0.002), was linked to poorer survival.

**Conclusion:**

The study highlights the significant roles of PTHrP and ezrin in breast cancer progression. Elevated levels of these proteins are associated with more aggressive disease, suggesting their capability as prognostic indicators and treatment targets in breast cancer. Additional studies are required to investigate their interaction and collective influence on breast cancer metastasis and treatment.

**Supplementary Information:**

The online version contains supplementary material available at 10.1186/s13000-025-01598-2.

## Introduction

Breast cancer remains one of the most common cancers globally, as reported by the GLOBOCAN 2020 data, ranking as the fifth leading cause of cancer-related mortality, with approximately 2.3 million new cases diagnosed annually [1]. In Egypt, the incidence of breast cancer is somewhat lower compared to the USA and Western nations, yet the mortality rate among Egyptian patients is notably higher [2].

Invasive breast carcinoma of no special type (IBC-NST) is the most frequently occurring among the different types of breast cancer, yet it presents a heterogeneous group with variable prognoses driven by diverse molecular characteristics that are not yet fully understood [3]. Identifying reliable biomarkers that predict therapeutic response and clinical outcome is crucial as treatment paradigms evolve.

Despite advances in early detection and management, the persistent issues of recurrence, metastasis, and therapeutic resistance underscore the imperative of advancing our understanding of the underlying molecular drivers of breast cancer [4, 5]. In this context, exploring the role of key molecular markers such as parathyroid hormone-related protein (PTHrP) and ezrin offers promising pathways. PTHrP and ezrin have emerged as potentially significant biomarkers in several cancers, including breast cancer [6–8].

Parathyroid hormone-related protein (PTHrP) is synthesized in both normal breast tissue and breast cancer cells, where it induces bone destruction when secreted by cancer cells metastasized to the bone. Upon release, PTHrP interacts with its receptor on adjacent bone cells. However, in the context of breast cancer, PTHrP does not follow this binding mechanism. Instead, it travels within the cells, exerting either tumor-promoting or tumor-suppressing effects [9].

Similarly, ezrin, a protein belonging to the ezrin-radixin-moesin (ERM) family, is pivotal in metastasis. It connects the cytoskeleton to the plasma membrane, thereby affecting cell adhesion, mobility, and the signaling pathways essential for tumor progression and regulation of tumor metastasis in several cancers, including breast cancer [7, 8].

Some studies suggest that both PTHrP and ezrin are not only overexpressed in various malignancies but also correlate with poor prognostic outcomes [6, 10]. However, their expression and prognostic value, specifically in IBC-NST, remain underexplored. This gap in knowledge presents a significant limitation for the stratification of patients and the tailoring of therapeutic strategies.

Interestingly, the interplay between PTHrP and ezrin in breast cancer has not been previously explored. This study investigates the interaction between PTHrP and ezrin within the specific setting of IBC-NST. By evaluating their immunohistochemical expression and correlation, this research aims to clarify their prognostic value in breast cancer.

## Patients and methods

This retrospective analysis was conducted on 160 archival paraffin-embedded tissue blocks collected from patients diagnosed with IBC-NST and normal breast tissue at the Menoufia University Hospital, Pathology Department, during the period from January 2018 to January 2022. Ethical clearance for this study was obtained from the Institutional Review Board (IRB) under code no (3/2022PATHO33).

The study group was divided into 123 cases of IBC-NST, treated primarily by modified radical mastectomy in 103 cases and conservative breast surgery in 20 cases, and 37 control cases comprising normal breast tissue samples obtained from reduction mammoplasty. Exclusion criteria included prior neoadjuvant therapy, other histological types rather than IBC-NST, and core biopsy specimens.

### Clinicopathological data

Clinicopathological data were gathered from medical records. Histopathological evaluations were performed on sections stained with hematoxylin and eosin. Tumor staging adhered to the most recent TNM classification guidelines set by the American Joint Committee on Cancer (AJCC) [11]. Tumors were categorized into early-stage (T1 and T2) and advanced-stage (T3 and T4). Grading was conducted using the Elston and Ellis grading system (1991) [12] and the Nottingham Prognostic Index was assessed [13].

Tumor-infiltrating lymphocytes (TILs) within the tumor microenvironment [14], the presence and extent of DCIS and perineural as well as lymphovascular invasion were evaluated [15, 16]. Mitotic figures were quantified as described by Lehr et al. (2012) [17]. The molecular classification was determined based on the immunostaining results for ER, PR, Her2neu, and Ki67 [18].

### Tissue microarray (TMA) blocks

For immunohistochemical analysis, tissue microarrays (TMAs) were created using a manual tissue arrayer (Breecher Instrument Manual Microarray, Wisconsin, USA). Each TMA block contained duplicate cores from each tissue sample, ensuring adequate sampling.

### Immunohistochemical analysis

The primary antibodies employed included a mouse monoclonal antibody for PTHrP (concentrated at 100 µl with a 1:100 dilution, Chongqing Biospes, Catalog # YMA1281) and a rabbit polyclonal antibody for ezrin (concentrated at 100 µl with a 1:150 dilution, ABclonal, Catalog # A19048), following an overnight incubation at dilutions recommended by the manufacturers. Standardized protocols using tris-EDTA buffer were employed for antigen retrieval, with specific controls (normal kidney for PTHrP and colon carcinoma for ezrin) in place for each staining batch [19–21].

Quantitative and qualitative assessments of immunohistochemical staining were systematically performed, evaluating both the staining patterns and intensities across samples. The semiquantitative H-score was utilized to integrate the proportion and intensity of staining, providing a composite expression score for each marker [22].

### Survival data analysis

Survival analyses utilized data from Menoufia University’s Clinical Oncology and Nuclear Medicine Department, spanning a follow-up period from January 2018 to January 2022. Kaplan-Meier and Hazard function curves were constructed to illustrate survival trends and associations [23].

### Statistical analysis

The data were managed and analyzed using IBM SPSS software version 20.0. (Armonk, NY: IBM Corp). Continuous data were reported as means and standard deviations (SD), whereas categorical data were represented as frequencies and percentages. Depending on suitability, categorical variables were compared using the Chi-square or Monto-Carlo tests. The Student’s t-test was applied for normally distributed quantitative variables, whereas the Kruskal-Wallis test was used for non-normally distributed quantitative variables. Survival analysis was conducted using the Kaplan-Meier method. The Cox proportional hazards model was used to identify independent prognostic factors. A *p*-value below 0.05 was deemed statistically significant.

## Results

### Clinicopathological data of breast cancer cases

The clinicopathological characteristics of the studied cases are detailed in Table 1.

**Table 1:** Clinicopathological data of the studied breast cancer cases (*n* = 123)

Clinical pathology		No.	%
**Age**	< 50	39	31.7
	≥ 50	84	68.3
	Min. – Max.	30.0–82.0	
	Mean ± SD.	55.05 ± 11.86	
	Median (IQR)	55.0 (47.0–65.0)	
**Menopausal status**	Premenopausal	55	44.7
	Postmenopausal	68	55.3
**Multifocality**	Unifocal	105	85.4
	Multifocal	18	14.6
**Grade**	1	2	1.6
	2	103	83.7
	3	18	14.6
**Size**	Min. – Max.	0.50–15.0	
	Mean ± SD.	3.73 ± 2.25	
	Median (IQR)	3.50 (2.0–5.0)	
**T stage**	T1	37	30.1
	T2	56	45.5
	T3	24	19.5
	T4	6	4.9
**T stage grouping**	Early	93	75.6
	Advanced	30	24.4
**N stage**	N0	27	22.0
	N1	29	23.6
	N2	34	27.6
	N3	33	26.8
**Nodal metastasis**	No	27	22.0
	Yes	96	78.0
**AJCC stage grouping**	Early	56	45.5
	Advanced	67	54.5
**Metastasis**	No	110	89.4
	Yes	13	10.6
**Bone metastasis**	No	116	94.3
	Yes	7	5.7
**In situ status**	Absent	92	74.8
	Present	31	25.2
**Grade of DCIS**	Low grade	9	7.3
	High grade	22	17.9
**DCIS Percent**	Non extensive	15	12.2
	Extensive	16	13.0
**Mitosis**	Min. – Max.	1.0–23.0	
	Mean ± SD.	3.87 ± 5.14	
	Median (IQR)	2.0 (1.0–3.0)	
**NPI group**	Good	18	14.6
	Moderate	49	39.8
	Poor	56	45.5
**NPI score**	Min. – Max.	2.40–8.0	
	Mean ± SD.	5.15 ± 1.28	
	Median (IQR)	5.30 (4.20–6.0)	
**TIL**	Low	75	61.0
	Moderate	26	21.1
	Dense	22	17.9
**LVI**	No	115	93.5
	Yes	8	6.5
**PNI**	No	114	92.7
	Yes	9	7.3
**Molecular subtype**	Luminal A	50	40.7
	Luminal B	47	38.2
	Triple negative	10	8.1
	Her2neu enriched	16	13.0
**ER**	Negative	26	21.1
	Positive	97	78.9
**PR**	Negative	37	30.1
	Positive	86	69.9
**Her2neu**	Negative	75	61.0
	Positive	48	39.0
**Ki 67 proliferative index**	Low proliferative index	70	56.9
	High proliferative index	53	43.1

### Immunohistochemical expression of PTHrP in the studied groups

**Fig. 1:**
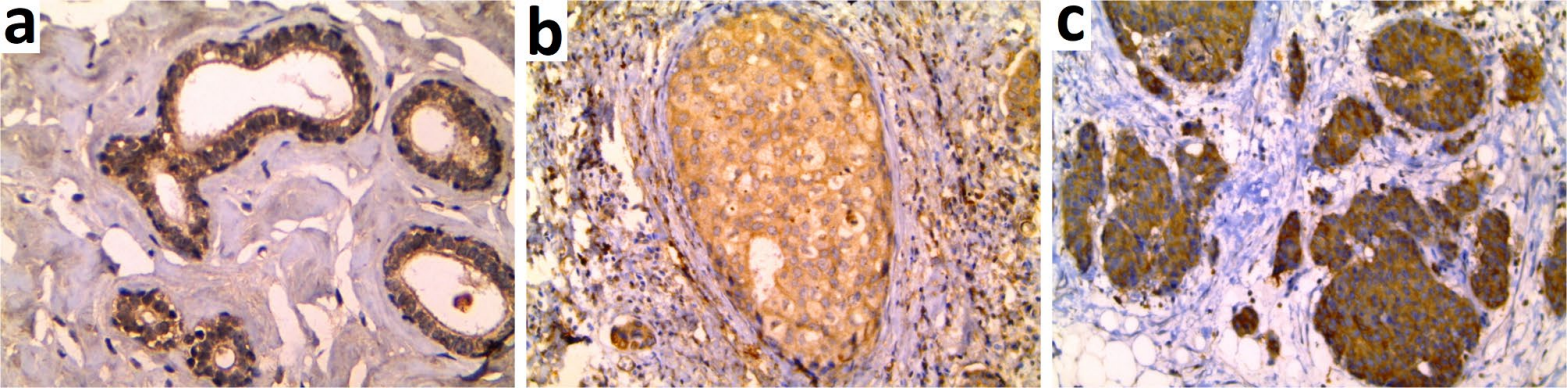
PTHrP Immunostaining showed **(A)** Mild cytoplasmic expression in normal breast lobules. **(B)** Moderate cytoplasmic expression in ductal carcinoma in situ (DCIS). **(C)** Strong cytoplasmic expression in invasive breast carcinoma (IBC) (IHC x200)

PTHrP was expressed in all investigated cases, either benign or malignant, as a cytoplasmic pattern of expression. There was a significant progressive increase in the percent of PTHrP expression from normal breast (mean ± SD of 64.86 ± 11.58, median of 65.0) to adjacent DCIS lesions (mean ± SD of 68.06 ± 7.92, median of 65.0) peaking in IBC-NST cases (mean ± SD of 80.53 ± 8.91, median of 80.0) (*p* < 0.001). Similarly, there was a progressive increase in PTHrP H score values from normal breast (mean ± SD 97.97 ± 37.18, median of 80.0) to adjacent DCIS lesions (mean ± SD 186.5 ± 36.77, median of 195.0) peaking in IBC-NST cases (mean ± SD 209.51 ± 49.86, median of 210.0) (*p* < 0.001). (Table 2; Fig. 1).

**Table 2:** Comparison of PTHrP expression in different studied groups

PTHrP		Control (*n* = 37)		Adjacent DCIS (*n* = 31)		IBC (*n* = 123)		Test of Sig. (*p*)
		No.	%	No.	%	No.	%	
**Percent**	Min. – Max.	40.0–85.0		60.0–90.0		60.0–95.0		H(p) = 64.716^*^ (< 0.001^*^)
	Mean ± SD.	64.86 ± 11.58		68.06 ± 7.92		80.53 ± 8.91		
	Median (IQR)	65.0 (60.0–70.0)		65.0 (60.0–70.0)		80.0 (70.0–90.0)		
**H score**	Min. – Max.	50.0–170.0		120.0–270.0		70.0–270.0		H(p) = 77.737^*^ (< 0.001^*^)
	Mean ± SD.	97.97 ± 37.18		186.5 ± 36.77		209.51 ± 49.86		
	Median (IQR)	80.0 (70.0–120.0)		195.0 (170.0–210.0)		210.0 (180.0–255.0)		

IQR: **Inter quartile range** SD: **Standard deviation H: H for Kruskal Wallis test**

p: *p* value for comparing different studied groups

*: Statistically significant at *p* ≤ 0.05

### Relationship between PTHrP expression and clinicopathological data in breast cancer tissues

**Fig. 2:**
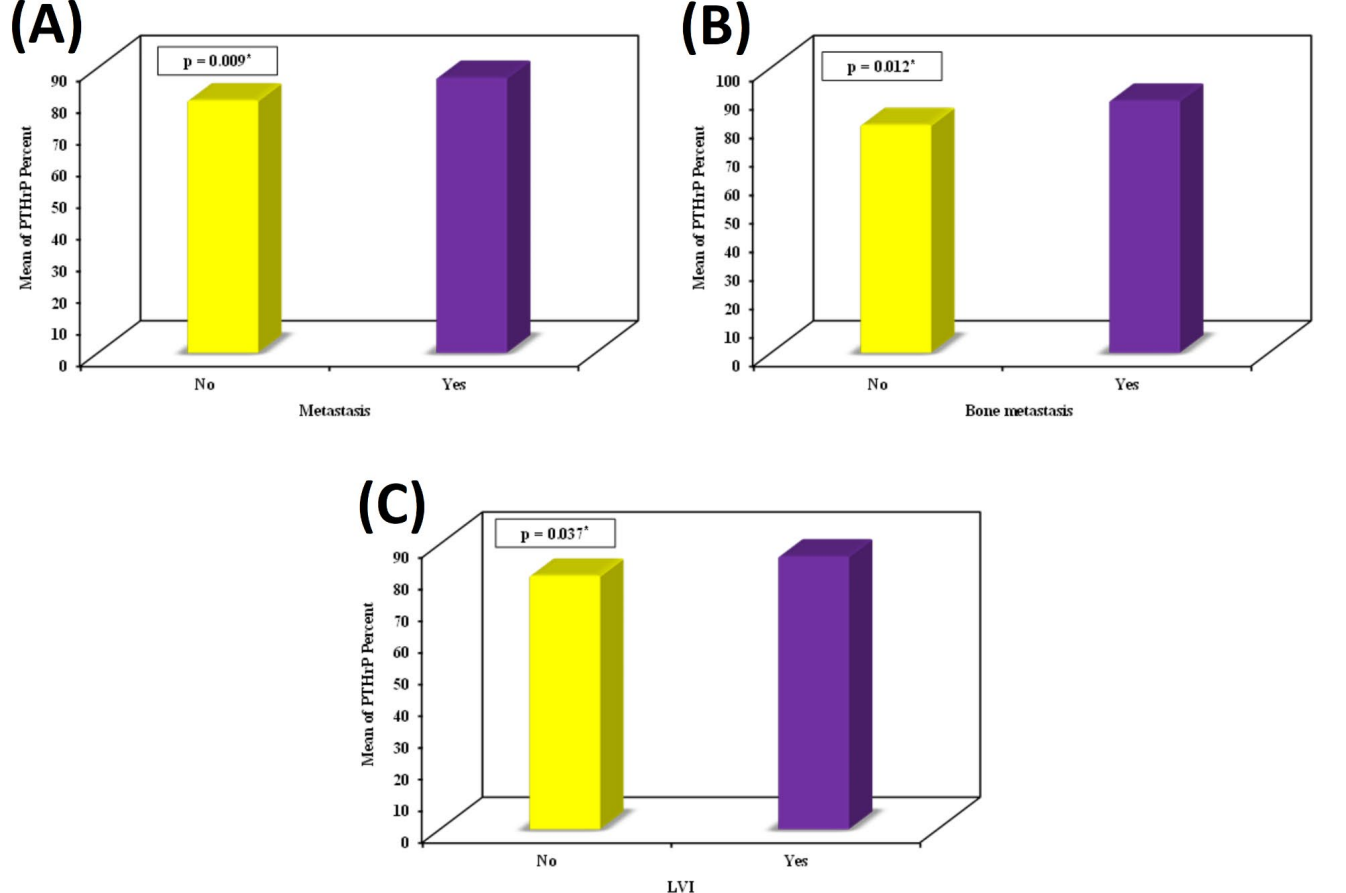
High PTHrP percent of expression was significantly associated with **(A)** metastasis (*p* = 0.009), **(B)** bone metastasis (*p* = 0.012) and **(C)** lymphovascular invasion (*p* = 0.037)

**Fig. 3:**
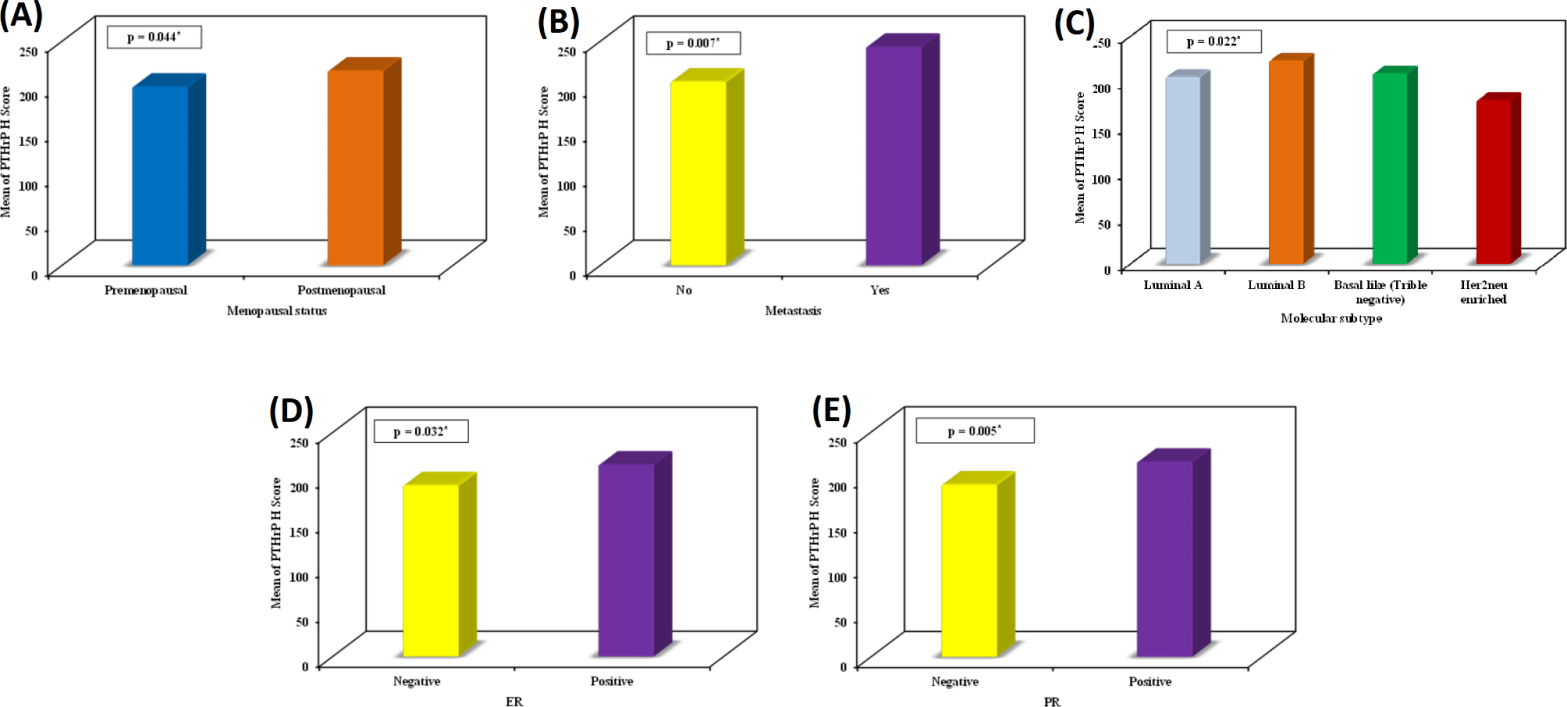
High PTHrP H score values were significantly associated with **(A)** postmenopausal status (*p* = 0.044), **(B)** presence of metastasis (*p* = 0.007), **(C)** luminal B subtype (*p* = 0.022), **(D)** ER positivity (*p* = 0.032), and **(E)** PR positivity (*p* = 0.005)

In IBC cases, high PTHrP percent expression was significantly associated with the presence of metastasis (*p* = 0.009), bone metastasis (*p* = 0.012), and lymphovascular invasion (*p* = 0.037) (Fig. 2). High PTHrP H score values were significantly associated with postmenopausal status (*p* = 0.044), presence of metastasis (*p* = 0.007), luminal B subtype (*p* = 0.022), ER positivity (*p* = 0.032), and PR positivity (*p* = 0.005) (Fig. 3).

### Immunohistochemical expression of ezrin in the studied groups

**Fig. 4:**
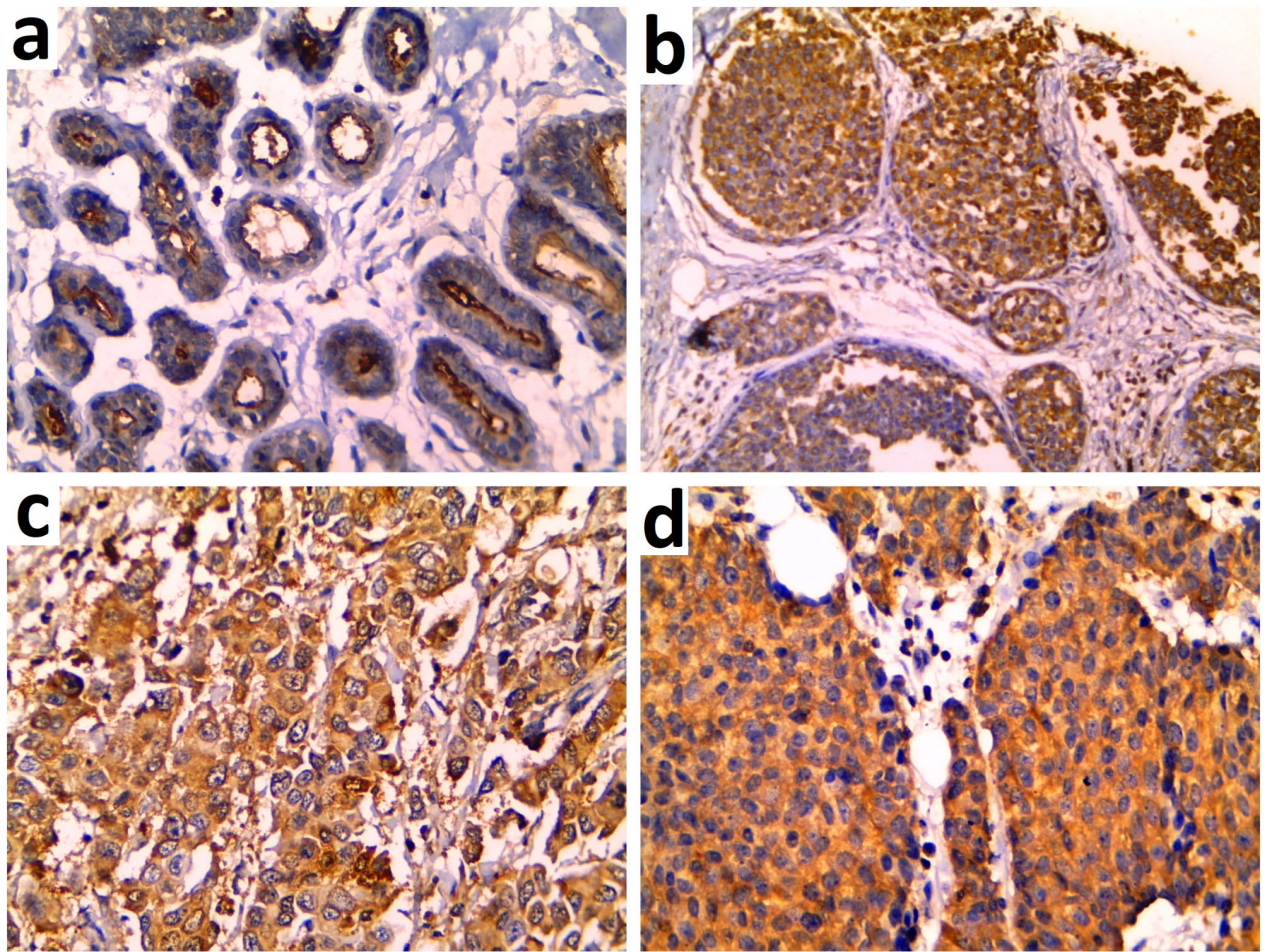
Ezrin Immunostaining showed **(A)** apical expression in normal breast lobules (IHC x200). **(B)** Ductal carcinoma in situ (DCIS) exhibited strong cytoplasmic expression of ezrin (IHC x100). **(C)** Invasive breast carcinoma (IBC) demonstrated moderate cytoplasmic expression of ezrin (IHC x400). **(D)** Invasive breast carcinoma (IBC) exhibited strong cytoplasmic expression of ezrin (IHC ×400)

Ezrin was expressed in 59.5% of the control group, all adjacent DCIS lesions, and 92.7% of the IBC-NST group as a cytoplasmic pattern of expression. There was a significant progressive increase in the percent of ezrin expression from the normal breast (mean ± SD of 67.05 ± 8.95, median of 70.0) to adjacent DCIS lesions (mean ± SD of 67.74 ± 8.25, median of 70.0) peaking in IBC-NST cases (mean ± SD of 79.25 ± 8.15, median of 80.0) (*p* < 0.001). Similarly, there was a progressive increase in ezrin H score values from normal breast (mean ± SD 75.23 ± 22.70, median of 70.0) to adjacent DCIS lesions (mean ± SD 157.4 ± 46.0, median of 160.0) peaking in IBC-NST cases (mean ± SD 200.4 ± 53.13, median of 210.0) (*p* < 0.001). (Table 3; Fig. 4).

**Table 3:** Comparison of ezrin expression in different studied groups

Ezrin		Control (*n* = 37)		Adjacent DCIS (*n* = 31)		IBC (*n* = 123)		Test of Sig. (*p*)
		No.	%	No.	%	No.	%	
**Expression**	Negative	15	40.5	0	0.0	9	7.3	χ^2^= 27.323^*^ (^MC^p (< 0.001^*^)
	Positive	22	59.5	31	100.0	114	92.7	
**Percent**	Min. – Max.	45.0–80.0		60.0–90.0		60.0–90.0		H = 48.930^*^ (< 0.001^*^)
	Mean ± SD.	67.05 ± 8.95		67.74 ± 8.25		79.25 ± 8.15		
	Median (IQR)	70.0 (60.0–75.0)		70.0 (60.0–70.0)		80.0 (70.0–90.0)		
**H score**	Min. – Max.	55.0–140.0		60.0–240.0		70.0–270.0		H = 61.786^*^ (< 0.001^*^)
	Mean ± SD.	75.23 ± 22.70		157.4 ± 46.0		200.4 ± 53.13		
	Median (IQR)	70.0 (60.0–80.0)		160.0 (130.0–180.0)		210.0 (160.0–240.0)		

IQR: **Inter quartile range** SD: **Standard deviation H: H for Kruskal Wallis test**

χ^2^: **Chi square test** MC: **Monte Carlo test**

p: *p* value for comparing different studied groups

*: Statistically significant at *p* ≤ 0.05

### Relationship between ezrin expression and clinicopathological data in breast cancer tissues

**Fig. 5:**
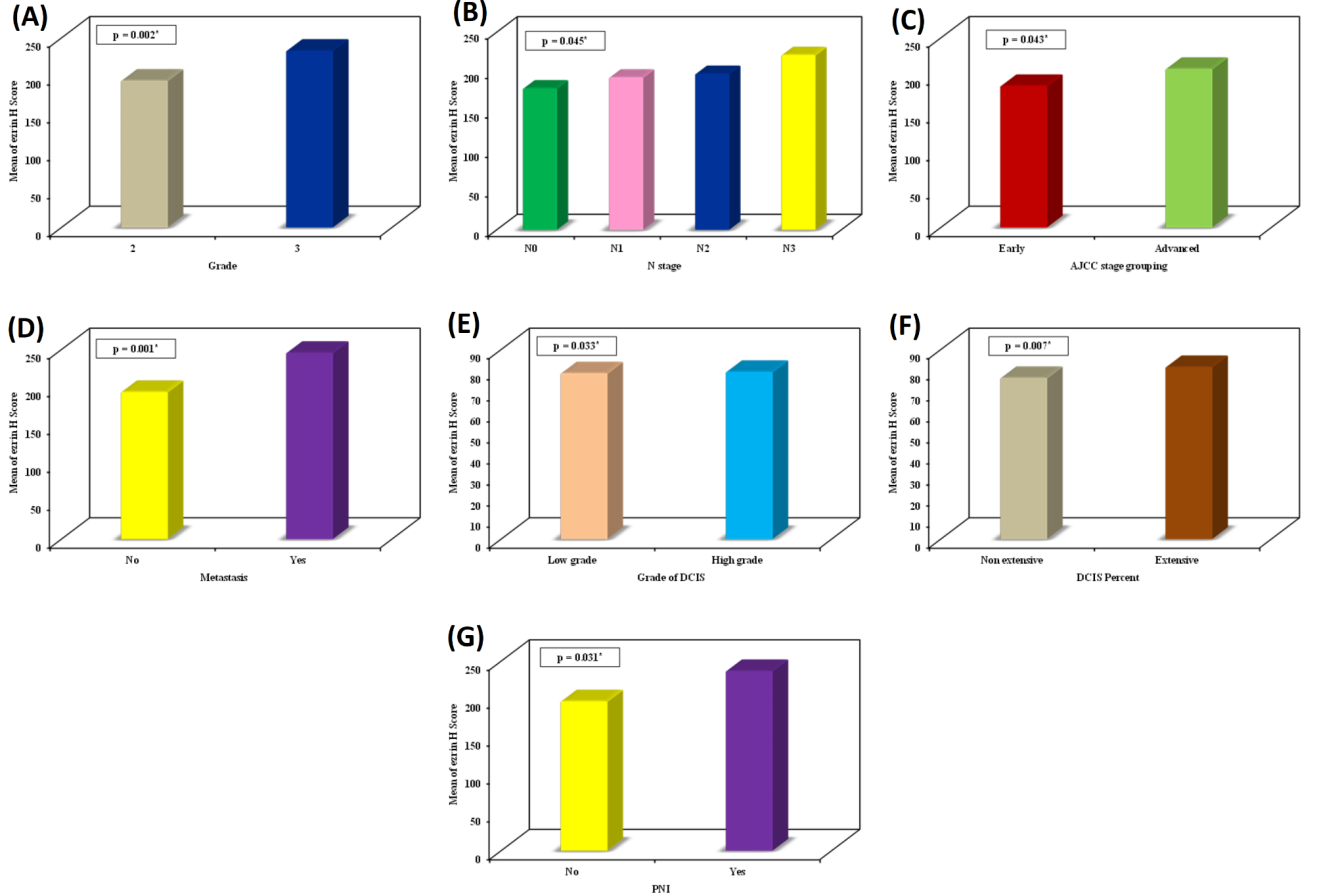
High ezrin H score values were significantly associated with **(A)** high tumor grade (*p* = 0.002), **(B)** high N stage (*p* = 0.045), **(C)** advanced AJCC stage (*p* = 0.043), **(D)** presence of metastasis (*p* = 0.001), **(E)** high grade of DCIS (*p* = 0.033), **(F)** extensive DCIS (0.007), and **(G)** perineural invasion (*p* = 0.031)

**Fig. 6:**
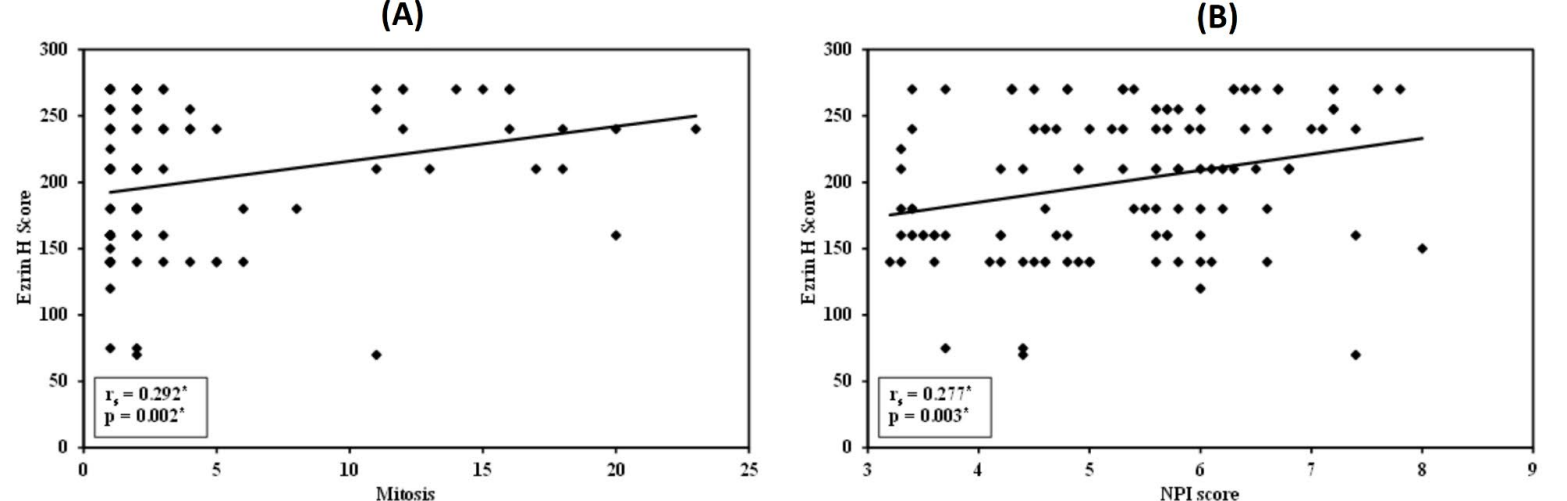
The correlation between ezrin H score and **(A)** high mitosis and **(B)** high NPI score

In IBC cases, elevated ezrin H score values were notably linked to high tumor grade (*p* = 0.002), high N stage (*p* = 0.045), advanced AJCC stage grouping (*p* = 0.043), presence of metastasis (*p* = 0.001), higher grade (*p* = 0.033), and a greater extent of DCIS and perineural invasion (*p* = 0.031) (Fig. 5). Additionally, there was a significant positive correlation between elevated ezrin H score and both increased mitotic activity (r_s_ = 0.292, *p* = 0.002) and higher NPI score (r_s_ = 0.277, *p* = 0.003) (Fig. 6). However, the correlation coefficients indicate that these are weak positive correlations.

### Relationship between PTHrP and ezrin expression in breast cancer tissues

**Fig. 7:**
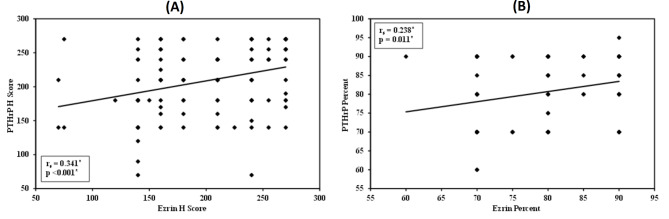
The correlation between PTHrP and ezrin regarding **(A)** percent and **(B)** H score

A significant direct correlation was observed between PTHrP and ezrin expression regarding percentage (r_s_ = 0.238, *p* = 0.011) and H score values (r_s_ = 0.341, *p* < 0.001) (Fig. 7). Although statistically significant, the correlation coefficients indicate a weak to correlation between these two markers.

### Overall survival analysis

**Fig. 8:**
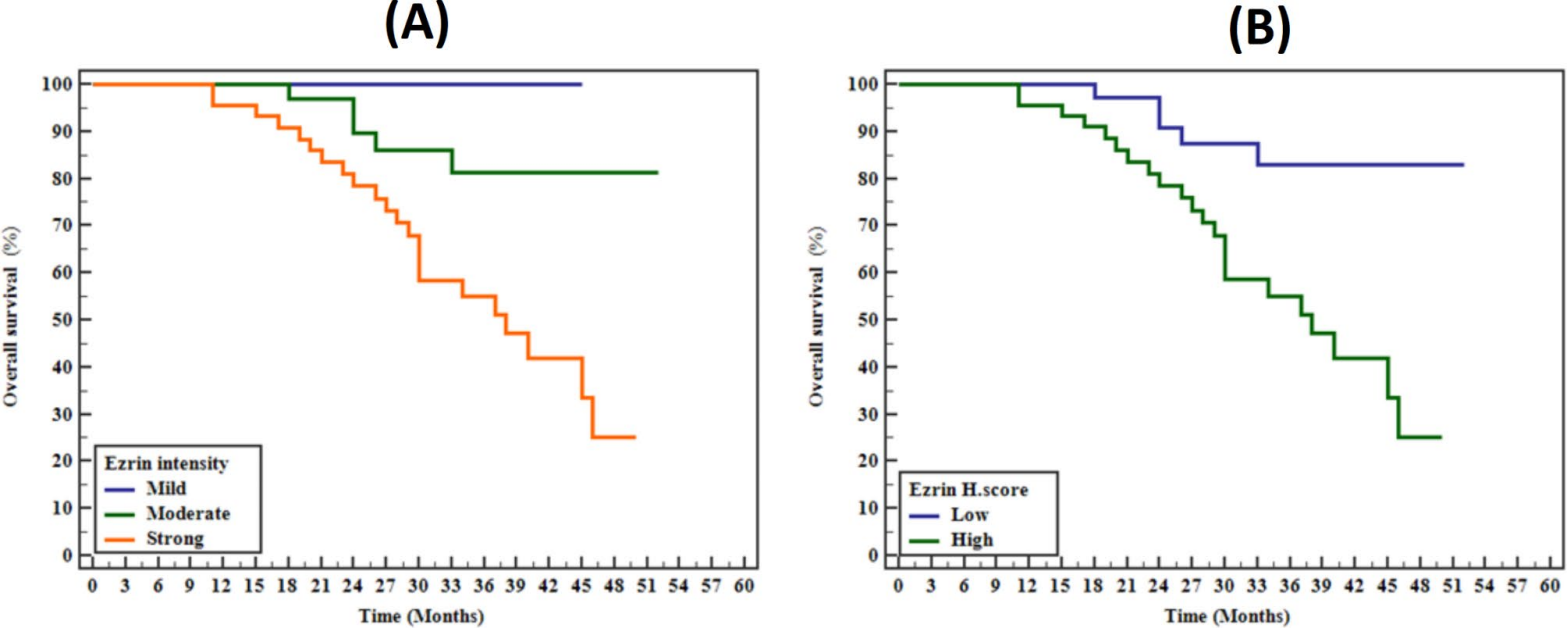
Univariate analysis of studied markers revealed prolonged OS with **(A)** moderate intensity of ezrin (*p* = 0.007) and **(B)** low ezrin H score (*p* = 0.002)

Overall survival data were available for 94 cases (76.4%). The follow-up period extended from January 2018 to December 2022, with survival time ranging from 9 to 52 months (mean ± SD: 30.15 ± 11.25 months, median: 30 months). During this period, 29 patients (23.6%) died of their disease. Prolonged overall survival was associated with moderate intensity (*p* = 0.007) and Low H score values (*p* = 0.002) of ezrin (Fig. 8). However, the Multivariate Cox regression analysis showed no significant ezrin expression independence in breast cancer patients’ overall survival.

## Discussion

Parathyroid hormone-related protein (PTHrP) is a recognized factor in breast cancer pathogenesis, particularly concerning bone metastasis [24]. The results of the current study revealed that PTHrP expression was significantly elevated in IBC cases compared to adjacent DCIS lesions and normal breast tissues. This finding aligns with previous studies that have shown elevated PTHrP levels in more aggressive breast cancer subtypes [6]. Specifically, PTHrP expression in the present study was associated with poor prognostic factors such as lymphovascular invasion, metastasis, and bone metastasis. These findings align with those of Yoshida et al. (2000) [25], who observed that elevated PTHrP expression is associated with the progression of breast tumors, the development of bone metastases, and reduced overall patient survival. Additionally, Powell et al. (1991) [26] reported a higher prevalence of PTHrP expression in breast cancer bone metastases compared to other metastatic sites.

PTHrP has been shown to promote angiogenesis at skeletal metastasis sites, which may aid in tumor cell spread, colonization, and growth [27–29]. Its enhancement of various angiogenic factors, such as IL-6, IL-8, CXCL1, and CCL2/MCP-1, likely contributes to the development and progression of tumors that produce PTHrP.

An additional explanation for PTHrP’s role in bone metastasis comes from an experimental model using the estrogen receptor-positive human MCF7 breast cancer cell line. These cells stayed dormant in bone after being injected into nude mice but started to aggressively colonize bone and form lytic deposits when PTHrP was overexpressed [30]. Gene expression analysis indicated that overexpression of PTHrP led to the downregulation of several pro-dormancy genes. This included LIFR and its downstream signaling target, SOCS3 [31].

Our study also found a significant association between high PTHrP H score values and positive hormone receptor status (ER and PR), luminal B subtype, and postmenopausal status. These findings are in line with the work of Henderson et al. (2001) [32] who reported that PTHrP expression was correlated with ER and PR positivity and better differentiation in breast cancer tissues. On the other hand, the study’s high PTHrP levels in primary tumors were associated with reduced bone metastases and better prognosis. This discrepancy might be due to differences in study design, study populations, methodologies, and the small dataset of the current study, highlighting the complexity of PTHrP’s role in breast cancer progression.

In literature, some studies [33, 34] reported that ER positivity was linked to a higher incidence and increased risk of bone metastasis. Another study connected PTHrP with ER and discovered that the expression of ER, OPNcyt, and PTHrPR1 proteins in primary breast cancers might be linked to a higher risk of bone metastasis [35]. Other studies [36] reported that the luminal subtype, particularly the luminal B subtype, was identified as the most prevalent phenotype among patients with bone metastasis.

The role of PTHrP in breast cancer remains complex, with its expression being influenced by various factors, including its actions on parathyroid hormone receptor (PTHR) and stage of tumorigenesis, which may affect its prognostic significance [9].

Regarding Ezrin expression, the results of this study revealed that it was significantly upregulated in IBC cases compared to adjacent DCIS lesions and normal tissues. High ezrin expression was associated with poor prognostic factors such as advanced AJCC stage, higher tumor grade, advanced N stage, and presence of metastasis and perineural invasion. These results align with the work of Ma et al. (2008) [37], who reported that increased ezrin expression was positively correlated with lymph node involvement, indicating that ezrin may serve as a biomarker for predicting lymphatic metastasis in breast carcinoma. Moreover, Xu et al. (2014) discovered that ezrin expression was linked to lymph node involvement and a high histological grade, corroborating our findings [38]. These findings are consistent with previous reports demonstrating a link between ezrin overexpression and increased metastatic potential in various cancer types [39, 40].

Our study further showed that ezrin expression was significantly correlated with high mitotic activity and the NPI score. However, these correlations were weak, as indicated by the low values of the correlation coefficients. This could be explained by the association of high ezrin expression with the metastatic potential of cancer rather than its role in promoting tumor proliferation.

The relocation of ezrin from the apical membrane in normal breast epithelial cells to the cytoplasm in invasive breast cancer cells suggests a functional role of ezrin in promoting tumor invasion and metastasis [20]. Ezrin is believed to facilitate breast cancer cell migration and invasion through the induction of EMT. Studies have demonstrated that silencing ezrin results in breast cancer cells adopting an epithelial morphology and losing their migratory ability. In contrast, overexpression of ezrin leads to a dispersed, spindle-shaped morphology. Additionally, ezrin-depleted cells exhibited higher levels of epithelial markers (E-cadherin and ZO-1) and lower levels of Vimentin, Snail, Slug, and MMP9, whereas the opposite pattern was observed in cells with ezrin overexpression [41].

Considering the significance of angiogenesis in cancer metastasis and progression [42], the role ezrin plays in breast cancer angiogenesis was previously investigated, and it was found that vascular mimicry and the microtubule formation ability of HUVECs decreased in cells with ezrin depletion but increased in cells with ezrin overexpression. Further Western blot analysis showed that silencing ezrin reduced the expression levels of VEGF and HIF1α, while overexpression of ezrin increased these levels, indicating that ezrin may possess pro-angiogenic properties in breast cancer [41].

Kaplan-Meier survival analysis in our study indicated that higher ezrin expression, both in terms of intensity and H score, was associated with poorer prognosis and reduced overall survival in breast cancer patients. These findings are supported by previous studies that have demonstrated the prognostic value of ezrin in predicting poor outcomes in breast cancer [39, 40].

Our study revealed a significant but weak correlation between PTHrP and ezrin expression in breast cancer tissue. Elevated ezrin levels were found to correlate with increased PTHrP levels, suggesting a possible interaction or joint regulation between these proteins. Although these findings indicate a potential interplay between PTHrP and ezrin, the weak correlation suggests that other mediators likely influence their expression or activity, highlighting the need for a larger sample size to validate this relationship. This co-expression could contribute to tumor growth and metastasis, thereby serving as important prognostic biomarkers and potential therapeutic targets in breast cancer therapy. Although the relationship between PTHrP and ezrin has not been extensively studied in breast cancer, similar interactions have been observed in other cancers, such as lung cancer bone metastases, where TGF-β induced both ezrin and PTHrP expression, facilitating tumor growth [43].

The co-expression of PTHrP and ezrin may indicate a synergistic role in enhancing breast cancer cells’ invasive and metastatic potential. Further research is needed to investigate whether the co-expression of PTHrP and ezrin has a synergistic or additive effect on tumor behavior and metastasis and to elucidate the underlying mechanisms of their interaction and their combined impact on breast cancer progression. Understanding these pathways could lead to developing novel therapeutic strategies targeting both proteins to inhibit metastasis and improve patient outcomes.

### Limitations of the study

This study has some limitations, including a relatively small sample size and a short follow-up duration, which may affect the generalizability of the results. Additionally, data on patient response to therapy prevents a comprehensive assessment of the therapeutic implications of PTHrP and ezrin expression.

## Conclusion

Our findings highlight the significant roles of PTHrP and ezrin in breast cancer progression and prognosis. Elevated expression levels of these proteins are associated with more aggressive disease. These biomarkers can potentially serve as valuable prognostic tools and therapeutic targets in breast cancer. Further studies are needed to fully understand the mechanisms underlying their co-regulation and their implications in breast cancer metastasis and treatment.

## Electronic supplementary material

Below is the link to the electronic supplementary material.

Supplementary Material 1

Supplementary Material 2

Supplementary Material 3

Supplementary Material 4

Supplementary Material 5

Supplementary Material 6

Supplementary Material 7

## Data Availability

No datasets were generated or analysed during the current study.

## Abbreviations

AJCC: American Joint Committee on Cancer
CI: Confidence interval
DCIS: Ductal Carcinoma In situ
ERM: Ezrin-Radixin-Moesin
IBC-NST: Invasive breast carcinoma of no special type
OS: Overall survival
PTHrP: Parathyroid hormone-related protein
TILs: Tumour-infiltrating lymphocytes
TMAs: Tissue microarrays
