# Supplementary figures and images for: Immunohistochemical expression of parathyroid hormone-related protein and ezrin in invasive breast carcinoma of no special type: a retrospective analysis
Source: Diagn Pathol. 2025 Jan 18;20:8. doi: 10.1186/s13000-025-01598-2 (PMC11742503; doi:10.1186/s13000-025-01598-2)

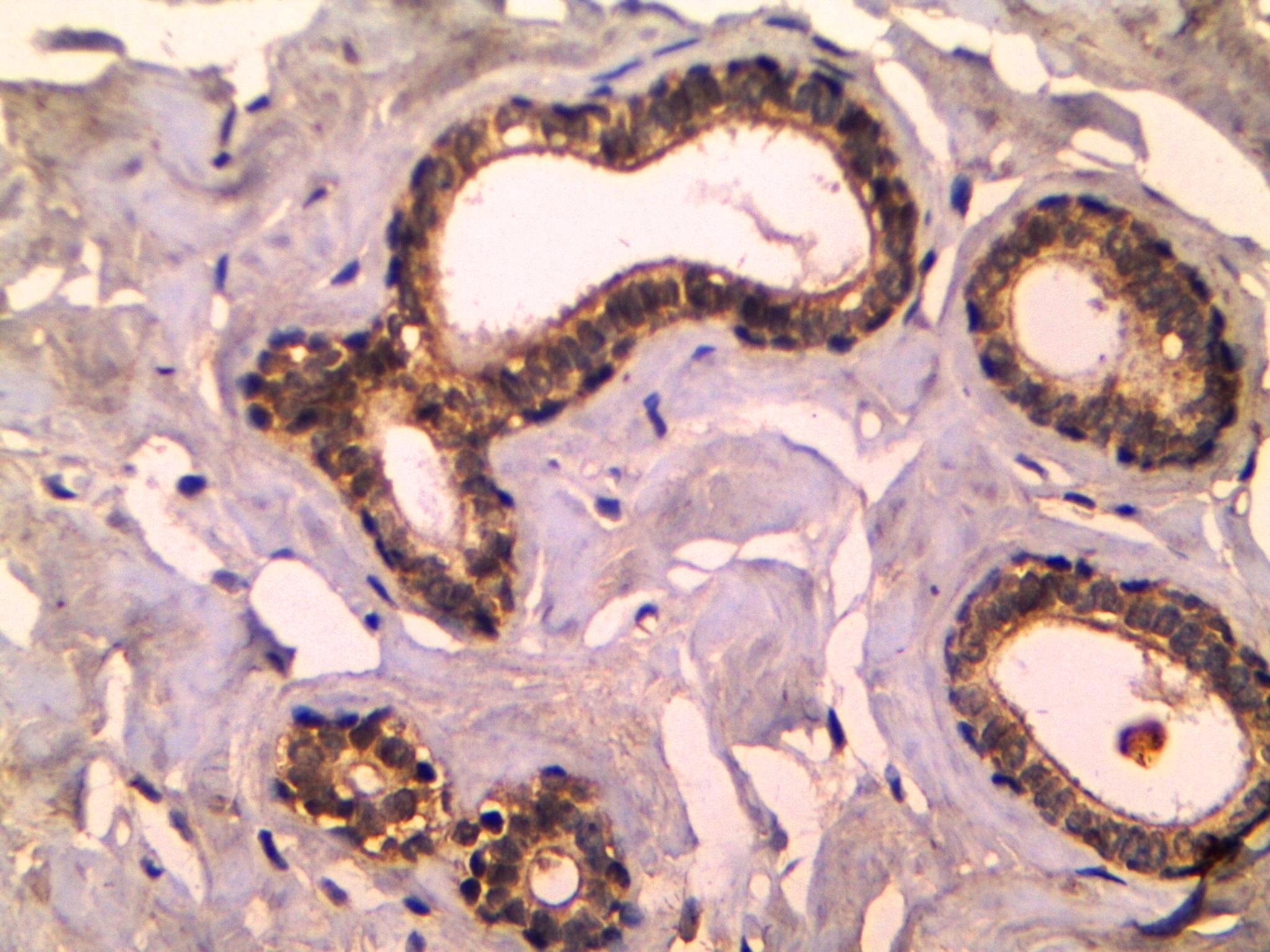

Supplement: Supplementary file 1 — Supplementary Material 1 [file 13000_2025_1598_MOESM1_ESM.jpg]

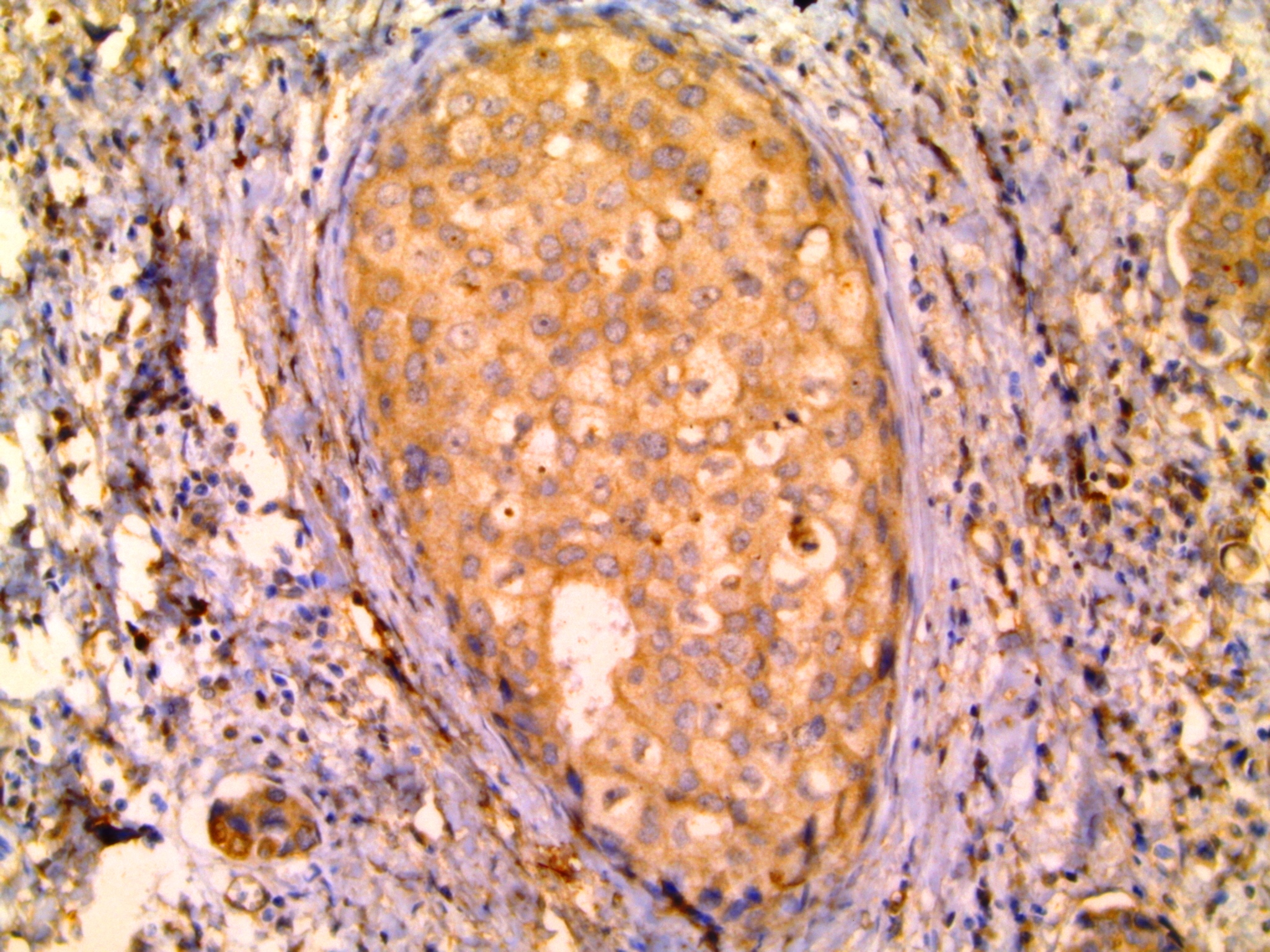

Supplement: Supplementary file 2 — Supplementary Material 2 [file 13000_2025_1598_MOESM2_ESM.jpg]

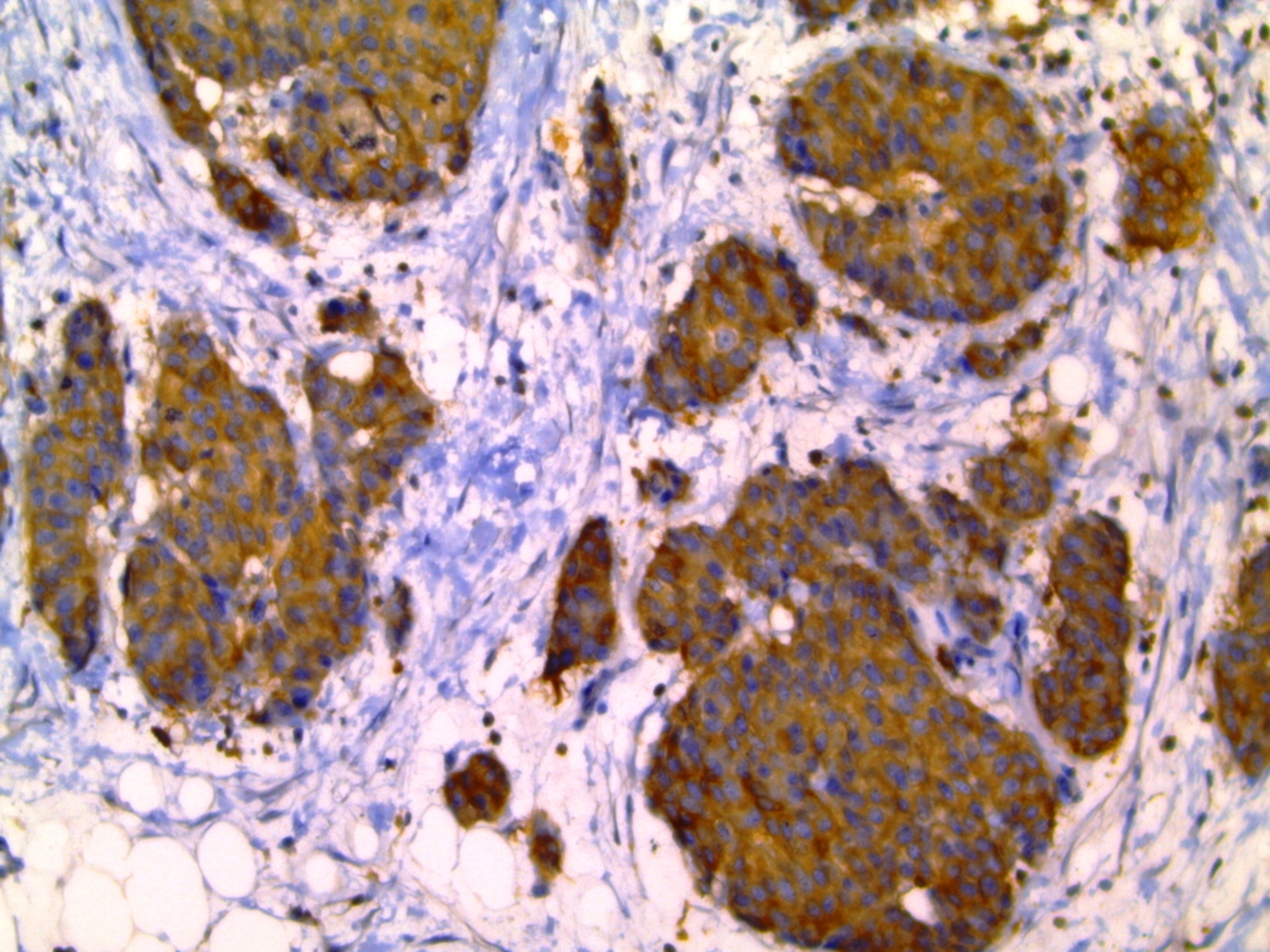

Supplement: Supplementary file 3 — Supplementary Material 3 [file 13000_2025_1598_MOESM3_ESM.jpg]

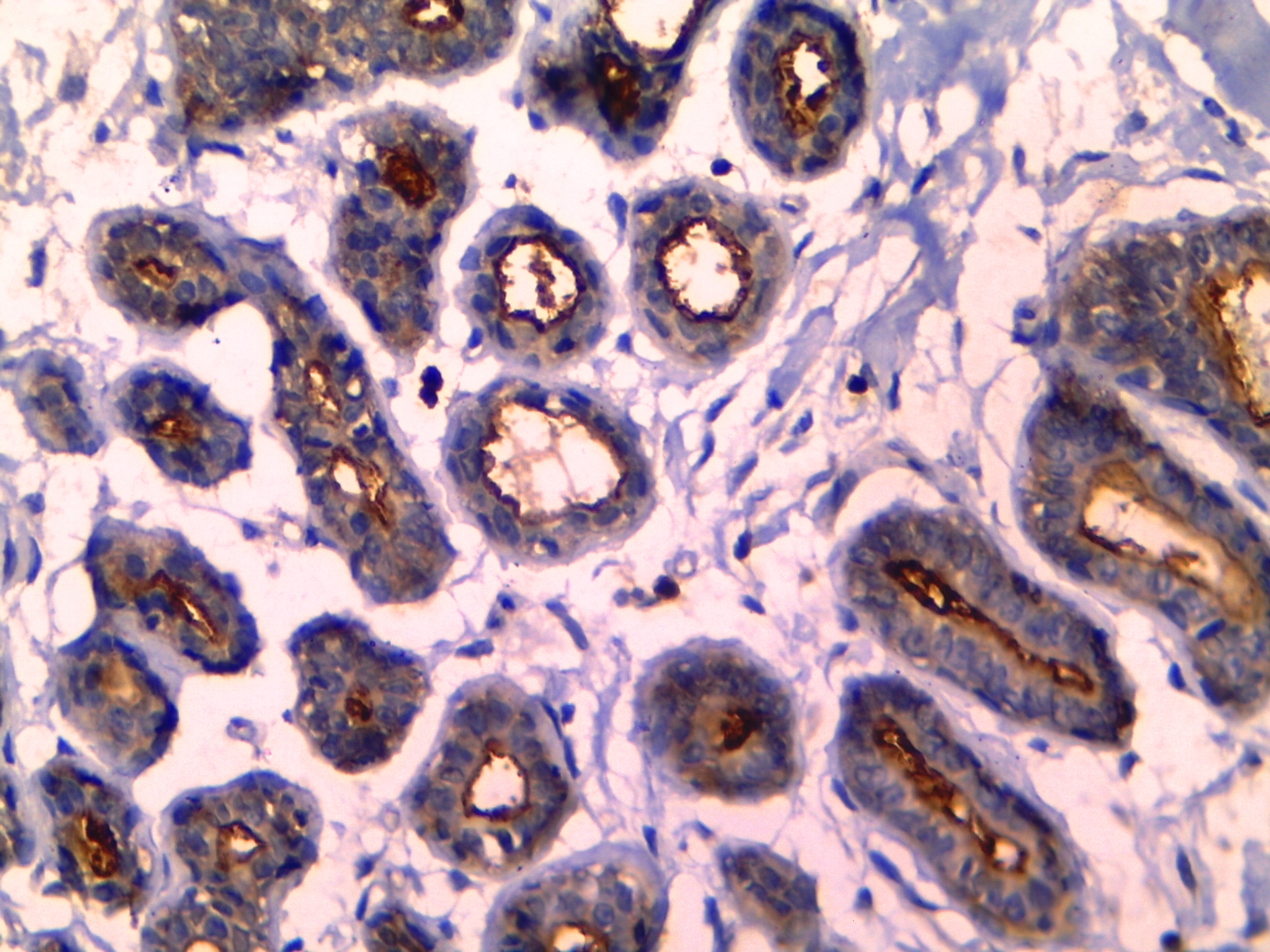

Supplement: Supplementary file 4 — Supplementary Material 4 [file 13000_2025_1598_MOESM4_ESM.jpg]

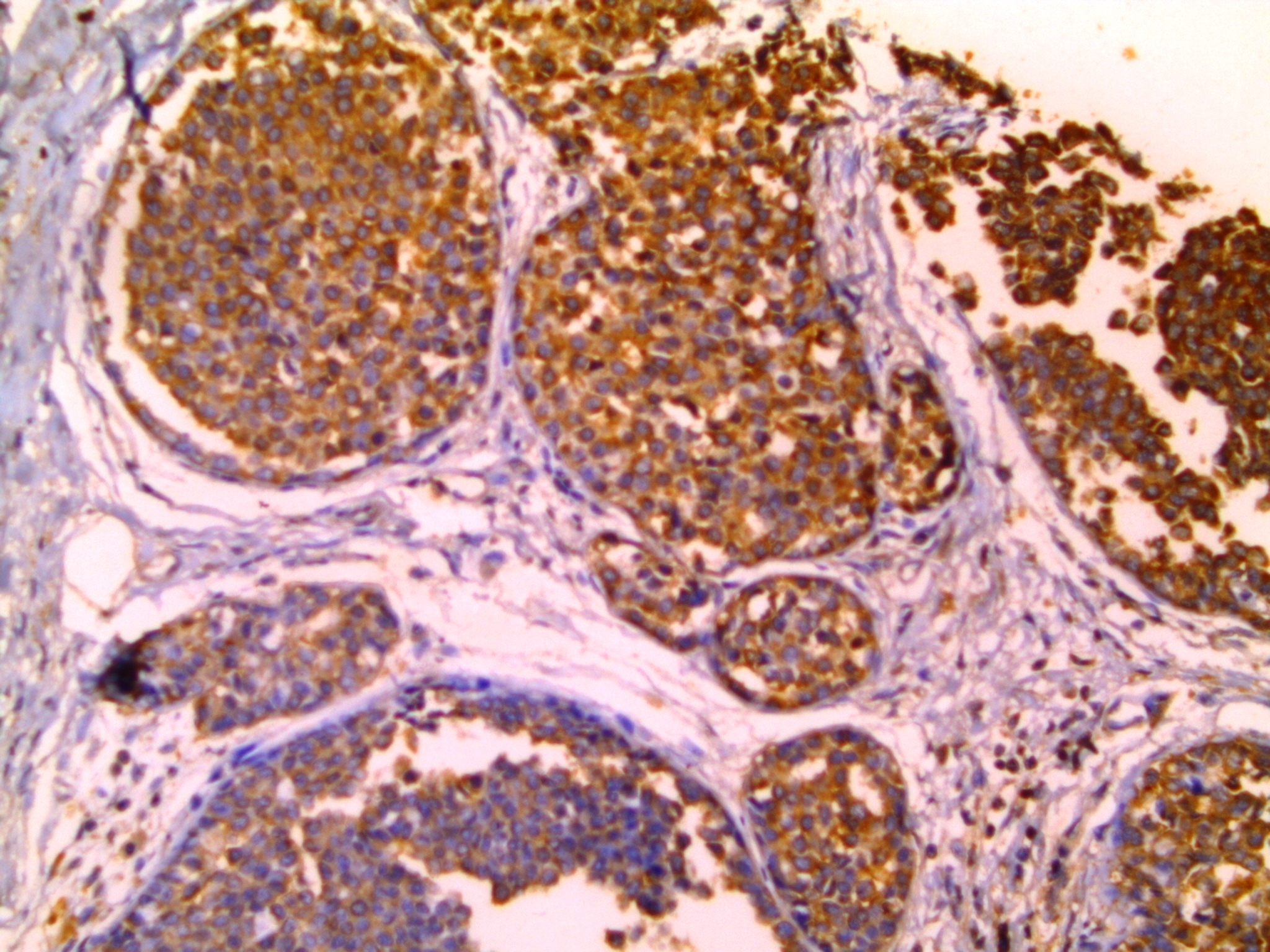

Supplement: Supplementary file 5 — Supplementary Material 5 [file 13000_2025_1598_MOESM5_ESM.jpg]

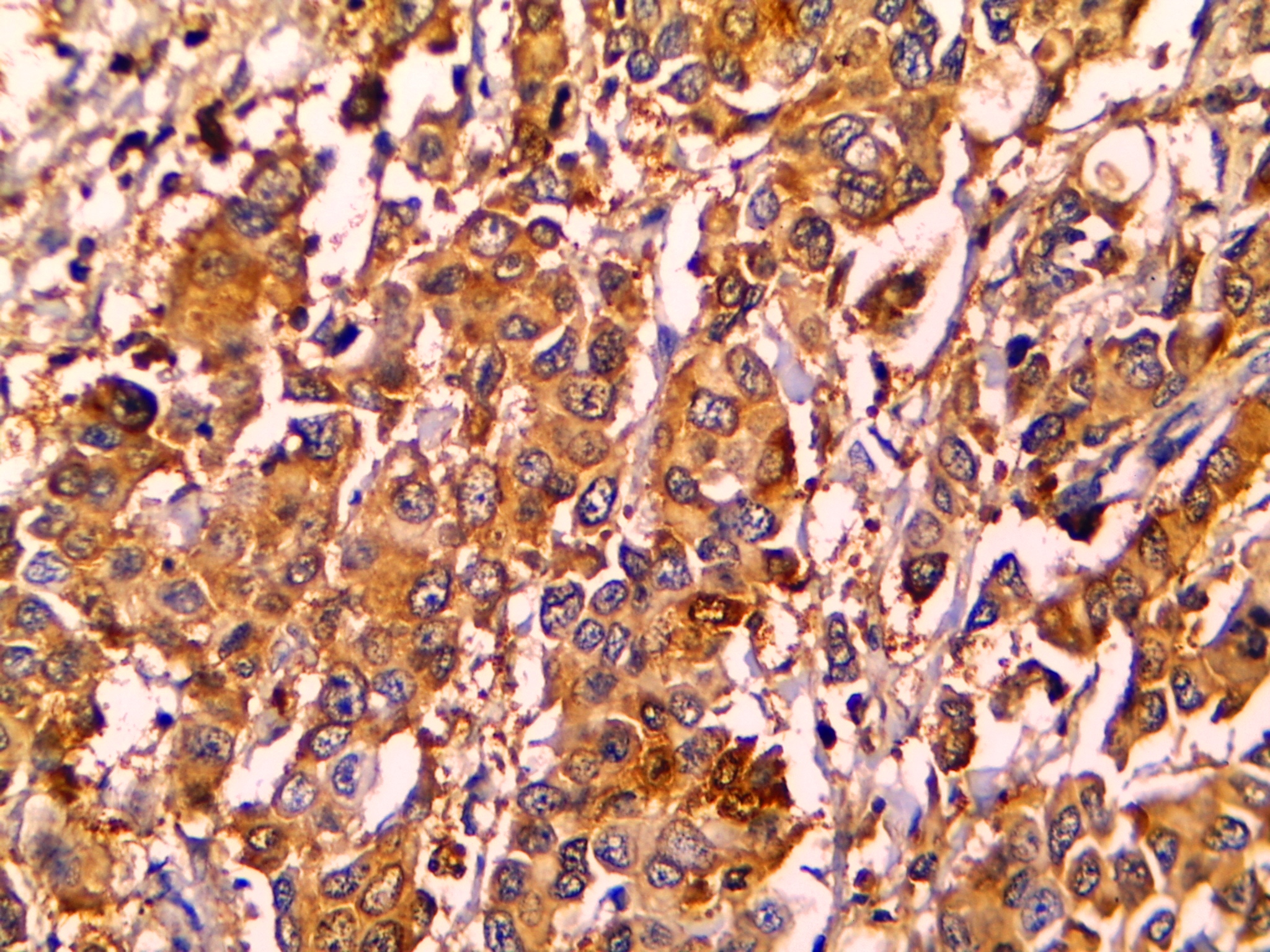

Supplement: Supplementary file 6 — Supplementary Material 6 [file 13000_2025_1598_MOESM6_ESM.jpg]

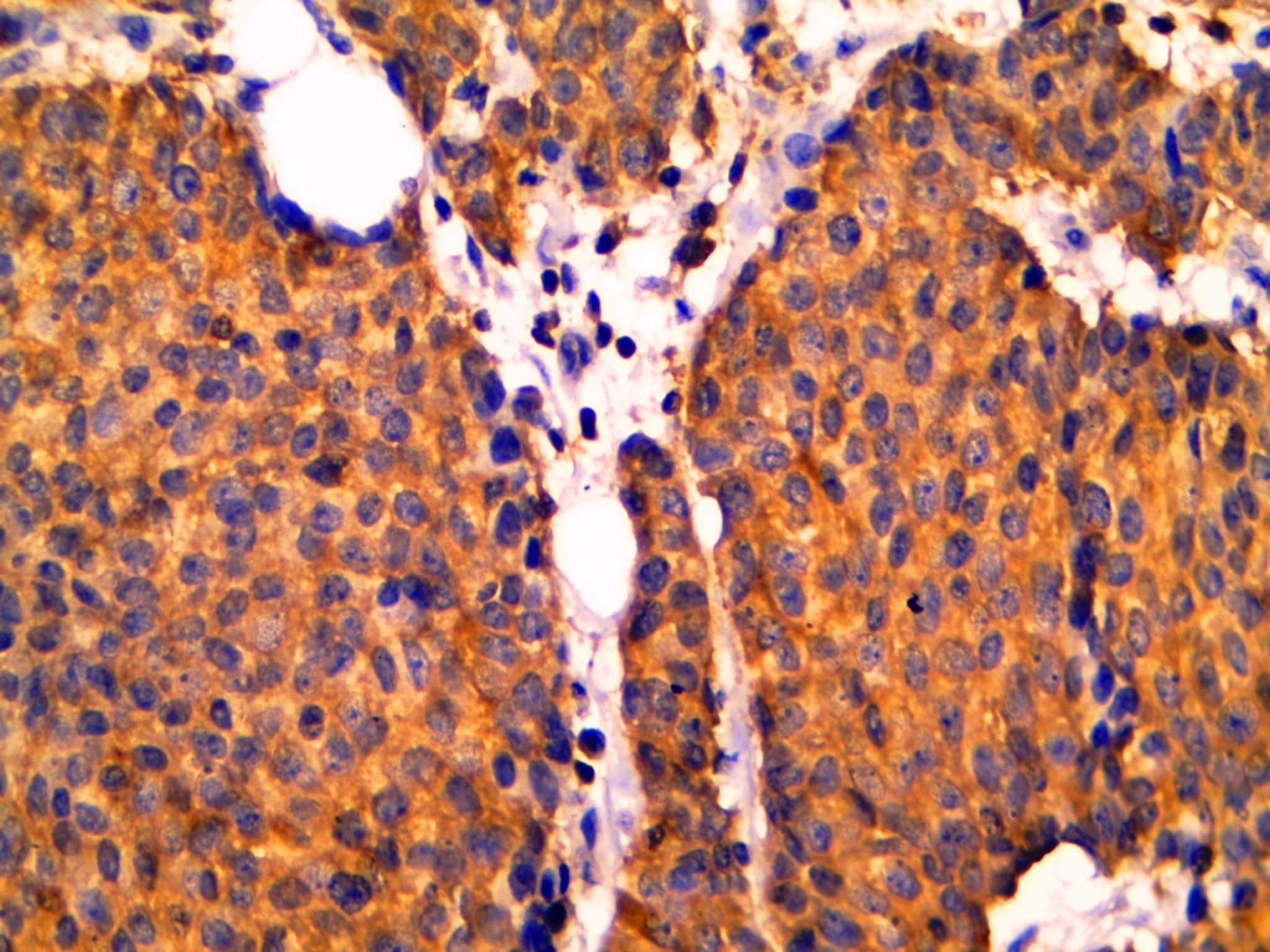

Supplement: Supplementary file 7 — Supplementary Material 7 [file 13000_2025_1598_MOESM7_ESM.jpg]
